# Olanzapine: An Antiemetic Option for Chemotherapy-Induced Nausea and Vomiting

**DOI:** 10.6004/jadpro.2014.5.1.8

**Published:** 2014-01-01

**Authors:** Megan V. Brafford, Ashley Glode

**Affiliations:** From Baptist Health Lexington, Lexington, Kentucky; Medical University of South Carolina, Charleston, South Carolina

## Abstract

Despite the appropriate use of pharmacologic and nonpharmacologic preventative measures, chemotherapy-induced nausea and vomiting (CINV) can be debilitating and can decrease quality of life for many patients. In addition, patients may be unwilling to continue chemotherapy treatment due to the uncontrollable nausea and vomiting associated with their therapy. Refractory CINV can occur at any point in a treatment cycle, despite adequate therapy for acute and delayed CINV. Current prevention strategies include using serotonin (5-HT3) receptor antagonists, corticosteroids, and/or neurokinin-1 receptor antagonists. Unfortunately, more pharmacologic options are needed to treat refractory CINV. The current standard of care for the treatment of refractory CINV includes phenothiazines, metoclopramide, butyrophenones, corticosteroids, cannabinoids, anticholinergics, and 5-HT3 receptor antagonists. Olanzapine, an atypical antipsychotic agent of the thiobenzodiazepine class, has the ability to target many different receptors, making it an attractive antiemetic agent.

Patients undergoing chemotherapy commonly experience nausea and vomiting, with nausea occurring in 60% of patients and vomiting occurring in 30% (Srivastava, Brito- Dellan, Davis, Leach, & Lagman, 2003). Chemotherapy-induced nausea and vomiting (CINV) can be debilitating for many patients and can decrease quality of life despite the administration of appropriate pharmacologic and nonpharmacologic methods of prevention. In addition, patients may be unwilling to continue chemotherapy treatment due to the uncontrollable nausea and vomiting associated with their therapy (Passik et al., 2002). As CINV is multifactorial, the treatment chosen should be patient-specific according to the cause. In addition to chemotherapy, other factors known to cause nausea and vomiting in patients with cancer include concomitant medications, tumor effect, hypercalcemia, vestibular dysfunction, central nervous system disorders, and visceral metastases (Srivastava et al., 2003; Passik et al., 2002).

The gastrointestinal (GI) tract, the chemoreceptor trigger zone (CTZ), and the vomiting center (VC) mediate emesis related to chemotherapy. Dopamine type 2 (D2), serotonin (5-HT2 and 5-HT3), substance P, serotonin muscarinic cholinergic (Ach), and histamine type 1 are the key neurotransmitters located in the CTZ and GI tract that are involved in the CINV response (Navari, 2009); see Table 1.

**Table 1:**
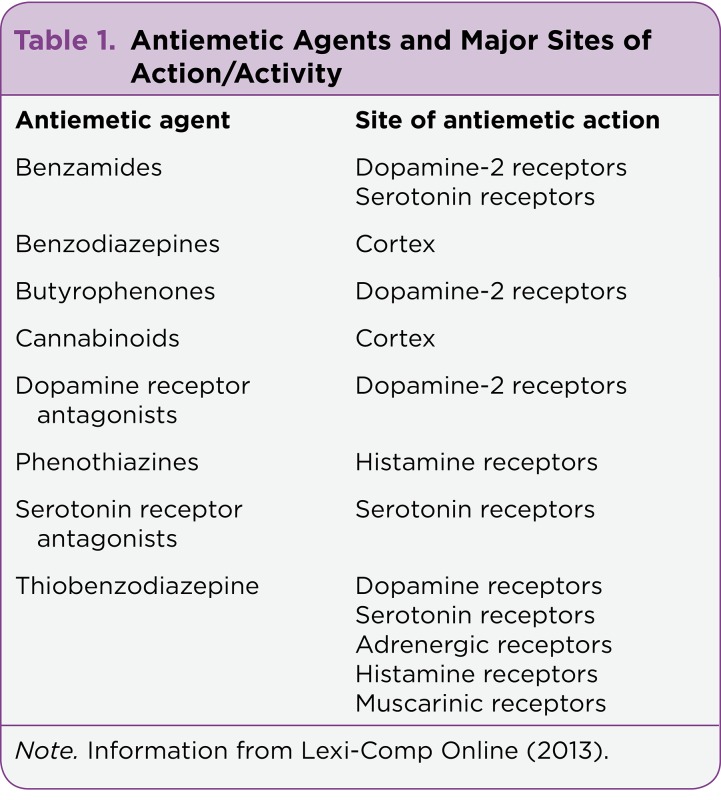
Table 1. Antiemetic Agents and Major Sites of Action/Activity

## Classification of CINV

Chemotherapy-induced nausea and vomiting can be further classified into four subtypes: acute, delayed, anticipatory, and refractory. Acute CINV occurs within 24 hours of receiving chemotherapy, whereas delayed CINV occurs most frequently 24 to 48 hours after chemotherapy but can occur up to 5 days post chemotherapy (Thompson & O’Bryant, 2010; Passik et al., 2004). Delayed CINV occurs in 50% to 70% of patients; however, these rates are decreasing with improved treatment and prevention (Passik et al., 2004). Treatment regimens for acute and delayed CINV are given prior to and after the chemotherapy regimen and may include a neurokinin-1 (NK1) receptor antagonist, a 5-HT3 receptor antagonist, and a corticosteroid, based on the level of emetogenicity.

Anticipatory CINV can occur hours to days before a patient receives chemotherapy. Pharmacologic treatment for this type of nausea and vomiting generally includes benzodiazepines, which help alleviate anxiety and the learned response to chemotherapy due to inadequate or ineffective premedication in prior cycles.

Refractory CINV can occur at any point in a treatment cycle, despite adequate therapy for acute and delayed CINV. If refractory CINV occurs, the emetic risk of the regimen should be reevaluated, and if possible, the therapy should be adjusted to include an agent with a different mechanism of action in future cycles. Refractory CINV can be treated by adding a scheduled antiemetic instead of using the antiemetic on an as-needed basis as well as assessing the patient for other potential causes of nausea and vomiting (Thompson & O’Bryant, 2010).

## Pharmacologic Options

Prophylactic antiemetics are given based upon the emetogenic risk of the agent(s) being given. Some regimens require no prophylaxis, whereas others require prophylaxis with medications from multiple classes. Current prevention strategies include using 5-HT3 receptor antagonists, corticosteroids, and/or NK1 receptor antagonists, which work well for the majority of patients, for most regimens. Unfortunately, some patients still experience CINV despite appropriate prophylaxis; therefore, more pharmacologic options are needed to treat refractory CINV.

The standard treatment options for refractory CINV currently include phenothiazines, metoclopramide, butyrophenones, corticosteroids, cannabinoids, anticholinergics, and 5-HT3 receptor antagonists. These medications may be combined to help alleviate nausea and vomiting (Srivastava et al., 2003). Common side effects associated with these agents include extrapyramidal symptoms (EPSs), restlessness, sedation, agitation, insomnia, and depression (Passik et al., 2002); see Table 2.

**Table 2:**
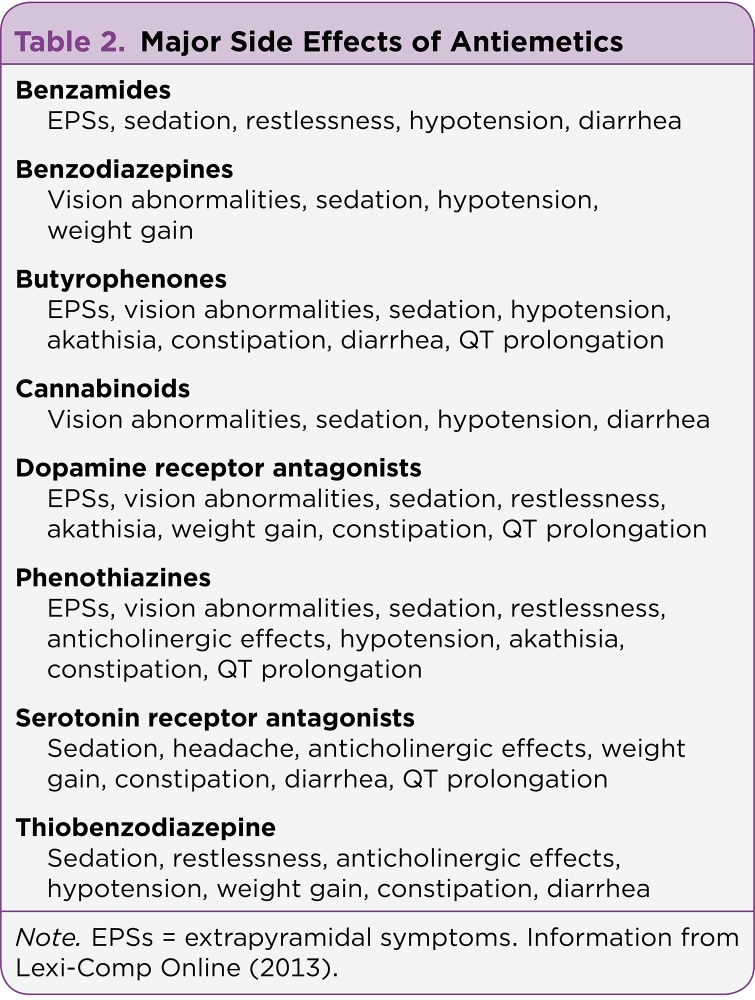
Table 2. Major Side Effects of Antiemetics

## Another Choice: Olanzapine

Olanzapine is an atypical antipsychotic agent of the thiobenzodiazepine class that has the ability to block many different receptors, which explains its antiemetic properties. Olanzapine targets dopaminergic (D1, D2, D3, D4), serotonergic (5-HT2A, 5-HT2C, 5-HT3, 5-HT6), adrenergic (á1), histaminergic (H1), and muscarinic (m1, m2, m3, m4) receptors. Olanzapine has a benefit over combination CINV regimens in that it can target multiple key receptors with one medication. The ease of once or twice daily administration and the use of a single agent can increase patient compliance in a setting where it may be difficult to take medications due to the nausea and vomiting (Srivastava et al., 2003).

The standard dosage of olanzapine for prophylaxis and treatment is 5 to 10 mg per day, with a maximum dose of 20 mg per day. Recommended starting doses are lower for women and elderly patients than for others. Olanzapine can also be given as a rescue dose: 5 mg every 4 hours on an as-needed basis.

Despite its advantages, there is an economic downside to olanzapine in comparison to other agents, due to recently approved generic options. There is a higher cost associated with olanza- pine than with the 5-HT3 receptor antagonists and combinations of phenothiazines and dronabinol (Navari et al., 2007; Lexi-Comp Online, 2013); see Tables 3 and 4. However, olanzapine is currently available in tablet, dissolvable disk, and parenteral formulations, which makes it a versatile antiemetic option (Srivastava et al., 2003).

**Table 3:**
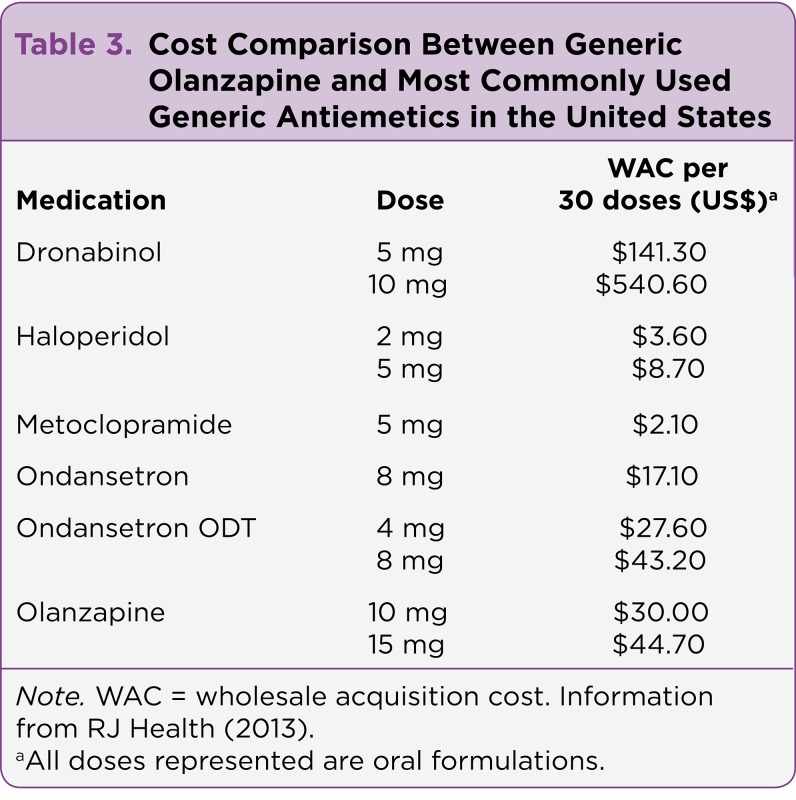
Table 3. Cost Comparison Between Generic Olanzapine and Most Commonly Used Generic Antiemetics in the United States

**Table 4:**
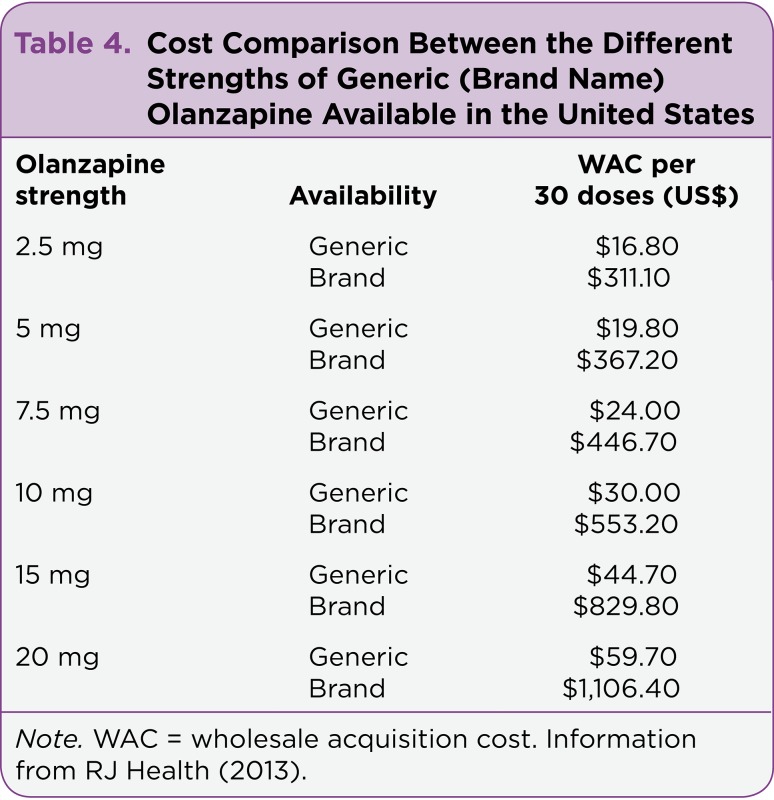
Table 4. Cost Comparison Between the Different Strengths of Generic (Brand Name) Olanzapine Available in the United States

## Clinical Evidence for Olanzapine

Several phase I and II studies (Navari et al., 2005; Navari et al., 2007; Tan et al., 2009) concluded that olanzapine can improve complete response rates for delayed CINV in patients receiving highly and moderately emetogenic chemotherapy as well as improve the quality of life of cancer patients during chemotherapy administration (Eaby-Sandy & Sherry, 2011).

An open-label pilot study showed that the level of nausea substantially decreased with each increase in dose, which may suggest a dose-response relationship; the 5-mg dose showed a statistically significant improvement in overall quality of life over baseline (*p* < .005; Passik et al., 2002). None of the doses of olanzapine was associated with increased EPSs compared with baseline (Srivastava et al., 2003).

A four-cohort, dose escalation, phase I trial showed that the maximum recommended dose based on results from the trial was 5 mg before chemotherapy and 10 mg post chemotherapy. The dose-limiting toxicities observed were grade 3 depressed level of consciousness and grade 3 fatigue. Other reported toxicities included nausea, anorexia, constipation, and mood alteration. This study showed that olanzapine is beneficial in preventing delayed CINV in patients receiving moderately emetogenic chemotherapy and that it may be used in patients receiving highly emetogenic chemotherapy (Passik et al., 2004).

Two phase II trials showed olanzapine 10 mg administered the day of chemotherapy and continued post chemotherapy is effective in preventing nausea and vomiting during the acute and delayed phases (Navari et al., 2005; Navari et al., 2007). Patients who received highly emetogenic chemotherapy (cisplatin 70 mg/m^2^) experienced no vomiting 24 hours post chemotherapy and no nausea during the entire period. Only 20% of patients experienced vomiting during the delayed phase (2 to 5 days post chemotherapy). During the acute phase, no patients experienced vomiting after moderately emetogenic chemotherapy (doxorubicin 50 mg/m^2^), and 85% of patients had no nausea. Approximately 15% of patients experienced vomiting during the delayed phase, and only 35% of patients experienced nausea (Navari et al., 2005). There was 100% complete response for day 1 (no emesis, no rescue needed), regardless of the emetogenicity of chemotherapy used in this trial. No vomiting was observed in 100% of patients in the acute phase and in 75% in the delayed phase. No patients experienced nausea during the acute period, and 50% of patients experienced nausea during the delayed period (Navari et al., 2007).

In a phase III trial, chemotherapy-naive patients were randomized to receive olanzapine 5 mg 2 days prior to chemotherapy and 10 mg on the day of chemotherapy and 3 days after chemotherapy or aprepitant 125 mg PO on day 1 followed by 80 mg PO on days 2 and 3. Olanzapine or aprepitant was administered in addition to palonosetron 0.25 mg IV on day 1 and dexamethasone 12 mg IV on day 1 and 4 mg PO twice daily on days 2 through 4.

During the highly emetogenic chemotherapy regimens (doxorubicin and cyclophosphamide [AC]; cisplatin; doxorubicin, bleomycin, vinblastine, dacarbazine [ABVD]; or ifosfamide), 87.5% of olanzapine patients and 77.8% of aprepitant patients experienced no anticipatory vomiting. In the 24 hours post chemotherapy, 75% of the olanzapine patients and 44% of the aprepitant patients experienced no vomiting. Only 62.5% of the olanzapine patients and 55.6% of the aprepitant patients experienced vomiting during the delayed phase (2 to 4 days post chemotherapy).

Rates of nausea were 25% for olanzapine patients vs. 22% for aprepitant patients during the anticipatory period; 63% for olanzapine patients vs. 44% for aprepitant patients during the acute period; and 63% for olanzapine patients vs. 67% for aprepitant patients during the delayed period (Shumway, Terrazzino, & Jones, 2009).

In a phase III trial, chemotherapy-naive patients were randomized to receive olanzapine 10 mg PO, palonosetron 0.25 mg IV, and dexamethasone 20 mg IV on day 1 followed by olanzapine 10 mg days 2 through 4 post chemotherapy or aprepitant 125 mg PO, palonosetron 0.25 mg IV, and dexamethasone 12 mg IV day 1 followed by aprepitant 80 mg PO and dexamethasone 4 mg PO twice daily days 2 through 4. The patients received cisplatin 70 mg/m^2^ or cyclophosphamide 500 mg/m^2^ and doxorubicin 50 mg/m^2^.

In the olanzapine group, no vomiting occurred and no rescue was needed in 97% of patients during the acute period, in 77% during the delayed period, and in 77% for the overall period. In the aprepitant group, no vomiting occurred and no rescue was needed in 87% of the patients in the acute period, in 73% in the delayed period, and in 73% for the overall period. No nausea was experienced in 87% of patients during the acute phase, in 69% during the delayed phase, and in 69% overall in the olanzapine group, compared with 87% during the acute phase, 28% during the delayed phase, and 38% overall in the aprepitant group (Navari, Gray, & Kerr, 2011).

Patients in a phase II trial received moderate to highly emetogenic chemotherapy in addition to the standard antiemetic of ondansetron plus a corticosteroid and metoclopramide. If a patient experienced breakthrough vomiting for at least one episode despite standard prevention, olanzapine 5 mg PO every 12 hours for two doses was administered. In the following 24 hours, 60.8% of patients reported a complete response with no vomiting, and 17.4% of patients reported a partial response with one vomiting episode (Chanthawong et al., 2011).

Relief of breakthrough vomiting was further investigated in a phase III trial where patients were randomized to receive olanzapine 10 mg PO three times daily for 3 days or metoclopramide 10 mg PO three times daily for 3 days for breakthrough CINV, then monitored for 72 hours after taking the prescribed medication. The patients received highly emetogenic chemotherapy of either cisplatin 70 mg/m^2^ or cyclophosphamide 600 mg/m^2^ and doxorubicin 50 mg/m^2^, with prophylactic antiemetics of dexamethasone 12 mg IV, palonosetron 0.25 mg IV, and fosaprepitant 150 mg IV on day 1 and then dexamethasone 8 mg PO on days 2 through 4.

No vomiting occurred in 70% of the olanzapine patients in comparison to 31% of the metoclopramide patients. In the olanzapine group, 68% of patients experienced no nausea; in the metoclopramide group, 23% of patients experienced no nausea (Navari, Nagy, & Gray, 2013).

## Adverse Reactions and Drug-Drug Interactions

The most common side effects associated with olanzapine are tolerable and mild and include somnolence, postural hypotension, constipation, dizziness, fatigue, dyspepsia, restlessness, and weight gain (Passik et al., 2002; Chanthawong et al., 2011). Previous studies have shown that the incidence of EPSs with olanzapine is significantly reduced compared with other antipsychotics (Passik et al., 2002). This finding may be due to olanzapine’s having five times the affinity for 5-HT2 receptors than for D2 receptors, resulting in fewer EPSs. Weight gain and increased appetite are additional benefits in cachectic patients (Passik et al., 2002). In the three phase III trials, no grade 3 or 4 toxicities were reported for olanzapine (Shumway et al., 2009; Navari et al., 2011; Navari et al., 2013).

Olanzapine has few drug-drug interactions and a wide therapeutic index. Carbamazepine increases the clearance of olanzapine by induction of CYP1A2. Fluvoxamine inhibits CYP1A2, increasing olanzapine serum levels. Probenecid inhibits uridine diphosphoglucuronateglucosyltransferase and influences the disposition of olanzapine. Neuroleptic malignant syndrome has rarely been reported with olanzapine. Insulin-resistant diabetes can occur, and the use of olanzapine may be associated with a prolonged QTc interval. Olanzapine is associated with a lower seizure threshold, and caution should be used in certain patients. It may be used relatively safely in patients with renal and liver dysfunction without dose adjustment (Lexi-Comp Online, 2013).

## Current Guidelines

Olanzapine is included in the Multinational Association of Supportive Care in Cancer (MASCC) and National Comprehensive Cancer Network (NCCN) guidelines for the treatment of refractory and breakthrough emesis. Other agents with this indication are lorazepam, dronabinol, haloperidol, metoclopramide, scopolamine, and prochlorperazine. Olanzapine is a good option for patients with nausea refractory to butyrophenones or phenothiazines or those who have EPSs resulting from traditional antiemetics.

A key concept is ensuring that breakthrough CINV is prevented instead of treated. Thus, routine around-the-clock administration of antiemetics should be used, rather than relying on as-needed dosing. In refractory CINV, if optimal treatment has been given as prophylaxis, repeated dosing of the same agents is unlikely to be successful. The general principle is to add one agent from a different drug class for an as-needed indication, and combination therapy is more effective than single-agent therapy. Prior to beginning the next chemotherapy cycle, patients who experienced refractory CINV should have their antiemetic regimen revised. In addition, the oral route might need to be avoided due to ongoing vomiting (NCCN, 2012; Roila et al., 2010).

Dopamine receptor antagonists (metoclopramide, prochlorperazine, droperidol, and haloperidol) were the core of antiemetic therapy before the introduction of 5-HT3 receptor antagonists. Current guidelines recommend that dopamine receptor antagonists be reserved for patients intolerant of or refractory to 5-HT3 receptor antagonists, NK1 receptor antagonists, and corticosteroids. Because of the high level of blockade of the dopamine receptors, dopamine receptor antagonists can cause EPSs, which may limit the use of these agents. Benzodiazepines are effective accompaniments to antiemetic regimens to treat anxiety and reduce anticipatory CINV (NCCN, 2012; Roila et al., 2010). According to a recent publication, cannabinoids may also be recommended to be reserved for patients intolerant of or refractory to 5-HT3 receptor antagonists, NK1 receptor antagonists, and corticosteroids (Navari, 2009). Adequate hydration or fluid repletion should be considered with cannabinoids, as electrolyte abnormalities can occur (NCCN, 2012; Roila et al., 2010).

## Conclusion

Advanced practitioners in oncology are in a key position to manage CINV. Some of the major factors to consider when selecting an antiemetic include the effectiveness of prevention vs. treatment, control in both the acute and delayed periods, adverse effects, ease of use, and cost-effectiveness. When all of these factors are considered, olanzapine may be a good option for patients experiencing CINV.
